# Gut Akkermansia muciniphila ameliorates metabolic dysfunction-associated fatty liver disease by regulating the metabolism of L-aspartate via gut-liver axis

**DOI:** 10.1080/19490976.2021.1927633

**Published:** 2021-05-25

**Authors:** Yong Rao, Zhiqi Kuang, Chan Li, Shiyao Guo, Yaohao Xu, Dandan Zhao, Yutao Hu, Bingbing Song, Zhi Jiang, Zhenhuang Ge, Xiyuan Liu, Chengdao Li, Shuobin Chen, Jiming Ye, Zhishu Huang, Yongjun Lu

**Affiliations:** a School of Pharmaceutical Sciences, Guangdong Provincial Key Laboratory of New Drug Design and Evaluation, Sun Yat-sen University, Guangzhou, China; b Run Ze Laboratory for Gastrointestinal Microbiome Study, School of Life Sciences, Sun Yat-sen University, Guangzhou, China; c Biomedical Center of Sun Yat-sen University, Guangzhou, China; d Lipid Biology and Metabolic Disease Research Group, School of Health and Biomedical Sciences, RMIT University, Melbourne, Australia

**Keywords:** Metabolic-dysfunction associated fatty liver disease (MAFLD), *Akkermansia muciniphila*, L-aspartate, bile acid metabolism, lipid oxidation, gut-liver axis

## Abstract

The gut bacterium *Akkermansia muciniphila* has been increasingly recognized for its therapeutic potential in treating metabolic disorders, including obesity, diabetes, and metabolicdysfunction-associated fatty liver disease (MAFLD). However, its underlying mechanism involved in its well-known metabolic actions needs further evaluation. The present study explored the therapeutic effect and mechanism of *A. muciniphila* in intervening MAFLD by using a high-fat and high-cholesterol (HFC) diet induced obese mice model. Mice treated with *A. muciniphila* efficiently reversed MAFLD in the liver, such as hepatic steatosis, inflammatory, and liver injury. These therapeutic effects persisted after long-term drug withdrawal and were slightly weakened in the antibiotics-treated obese mice. *A. muciniphila* treatment efficiently increased mitochondrial oxidation and bile acid metabolism in the gut-liver axis, ameliorated oxidative stress-induced cell apoptosis in gut, leading to the reshaping of the gut microbiota composition. These metabolic improvements occurred with increased L-aspartate levels in the liver that transported from the gut. The administration of L-aspartate *in vitro* or in mice displayed the similar beneficial metabolic effects mentioned above and efficiently ameliorated MAFLD. Together, these data indicate that the anti-MAFLD activity of *A. muciniphila* correlated with lipid oxidation and improved gut–liver interactions through regulating the metabolism of L-aspartate. *A. muciniphila* could be a potential agent for clinical intervention in MAFLD.

## Introduction

Metabolicdysfunction-associated fatty liver disease (MAFLD, previously known as Nonalcohol fatty liver disease, NAFLD) is defined as a wide spectrum of liver diseases, including simple steatosis (NAFL), nonalcoholic steatohepatitis (NASH), fibrosis and cirrhosis, and even hepatocellular carcinoma (HCC).^1,2^ MAFLD has become a leading liver disease, with a prevalence of 22–29% in adults worldwide, greater than 75% of whom are overweight and obese.^3^ However, to date, no approved pharmacological agents are available for MAFLD treatment.

MAFLD is a metabolic syndrome characterized by heavy lipid accumulation, and associated with oxidative stress, lobular inflammation, apoptosis, and a certain degree of fibrosis progressively in clinical hepatic pathologic findings.^4^ Among these manifestations, hepatic steatosis serves as the first initiator (first hit), displaying aggressive injury to hepatocytes, and followed by multiple hits including oxidative stress, mitochondrial dysfunction and degeneration, and apoptosis, which lead to fibrosis in the liver.^5^ Our previous and recent studies have demonstrated that increasing hepatic lipid oxidation by pharmacologic treatment or gene editing is an efficacious approach to combat MAFLD by decreasing the lipid content and ameliorating progressive oxidation stress, lipotoxicity, inflammation and fibrosis,^6–8^ which agree with the recommendations of anti-NASH drug development.^9^

The gut microbiota is a virtual metabolic and endocrine organ that comprises approximately 10^14^ microbial cells inside the gut.^10^ These microorganisms are associated with the regulation of host metabolism, and immunity *via* their abundant metabolites, such as short-chain fatty acids (SCFAs) and amino acids.^11,12^ Recently, the gut microbiome has drawn extensive attention and served as a therapeutic approach in combating MAFLD.^13,14^ *Akkermansia muciniphila*, represents 1 to 5% of the microbial community in humans, and is considered to be a next-generation beneficial microbes.^15^ Treatment with *A. muciniphila* has been shown to protect metabolic disorders, including fat-mass gain, metabolic endotoxemia, adipose tissue inflammation, and insulin resistance.^16–18^ Also, several studies have revealed that *A. muciniphila* is an efficacious agent to prevent the worsening of metabolic disorders such as obesity.^16,18,19^ Additionally, several studies have shown the efficacious treatment of *A. muciniphila* in combating MAFLD *in vivo* by either direct treatment with *A. muciniphila* or enrichment of its abundance in gut through diet or drugs interventions .^20–22^ Although the beneficial metabolic effects of *A. muciniphila* have been well documented, however, to date, the detailed mechanism underlying the therapeutic ability of *A. muciniphila* is incompletely understood.

Therefore, the first aim of the present study was to evaluate the therapeutic properties of *A. muciniphila* in treating MAFLD in an obese mice model induced by high-fat and high-cholesterol (HFC) diets, which well mimics the humans daily diet and is critical for inducing hepatocyte injury and driving the development of fatty liver-associated disease.^23^ Our second aim was to investigate the molecular mode of action of *A. muciniphila* by identifying the significant metabolites correlated with MAFLD treatment. This was carried out by performing hepatic metabolomics analysis of the liver samples from *A. muciniphila*‐treated mice, in terms of the important pathological pathways leading to MAFLD and by relevant intervention studies in cell models. Our data revealed that mice treated with *A. muciniphila* efficiently increased lipid oxidation and bile acid metabolism, thus maintaining the integrity of the gut barrier and reshaping the gut microbiota composition. These beneficial metabolic effects occurred with increased levels of L-aspartate in the liver that transported from the gut. Moreover, gavage of L-aspartate in mice directly increased energy expenditure and ameliorated MAFLD.

## Results

### Gavage of *A. muciniphila* attenuated MAFLD in obese mice

Previous studies have shown that the gavage of *A. muciniphila* reshapes the gut microbiota composition in mice.^24,25^ In present study, we examined the gut microbiota composition in the feces of HFC mice with or without administration of *A. muciniphila*. Two distinguished clusters corresponding to the HFC mice and *A. muciniphila*-treated HFC mice revealed substantial differences in the microbial community structure between the groups (Figure S1A). Gavage of *A. muciniphila* in the mice increased the richness and diversity of gut microbiota by α-diversity analysis [indicated by observed operational taxonomic units (OTUs) and Shannon index] (Figure S1B) and improved the microbial community structure (Figure S1C-D). Notably, *A. muciniphila* was identified as one of the distinguished microbiota components by LEfSe analysis (*p* = .0096; Figure S1E).

**Figure 1.**
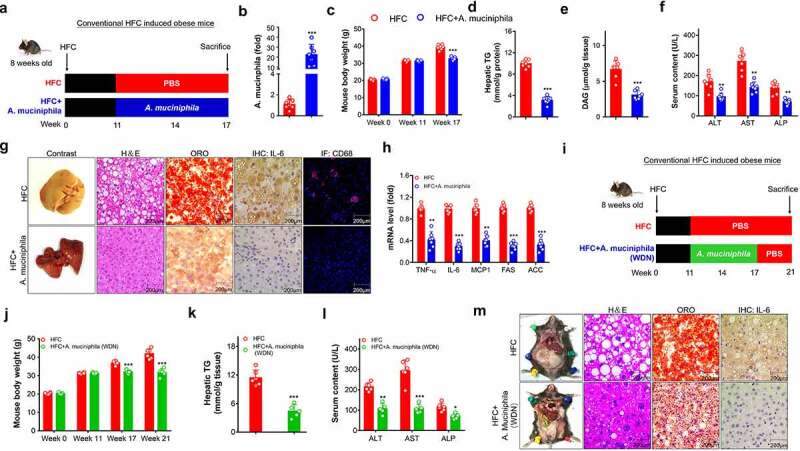
*A. muciniphila* attenuated obese MAFLD induced by HFC diet feeding. (a-h) Eight-week-old male C57BL/6 mice were fed with an HFC diet for 11 weeks to induce obese MAFLD. They were then randomly divided into two subgroups to treat with PBS as a control (HFC group) or *A. muciniphila* in PBS (HFC+*A. muciniphila*) orally every other day for 6 weeks. (a) Schematic diagram of *A. muciniphila* treatment. (b) Abundance of *A. muciniphila* in feces. (c) Mouse body weight at week 17. (d-e) Hepatic triglyceride (TG) and diacylglyceride (DAG) levels. (f) Plasma levels of AST, ALT and ALP. (g) Pathologic examination of the liver by hematoxylin ＆ eosin (H & E) staining, oil red O staining (ORO), and immunofluorescence examination of IL-6 and CD68. Representative images were captured. Scale bar, 200 μm. (h) Expression of mRNA markers for steatosis and inflammation markers. The levels of genes in the HFC control group were set as 1, and the relative fold increases were determined by comparison with the HFC control group. N = 5–8 mice/group. * *p* < .05, compared with HFC control group mice. (i-m) Sustainability of the anti-MAFLD efficacy of *A. muciniphila* after the cessation of its administration. (i) Schematic diagram of the experimental design. After 6 weeks of administration, *A. muciniphila* was withdrawn from the HFC-induced obese MAFLD mice followed by a period of 4 weeks of washout to evaluate of the sustainability of the anti-MAFLD efficacy. (j) Mouse body weight. (k) TG level in the liver. (l) Plasma levels of AST, ALT and ALP. (m) Pathologic examination of the liver by H ＆ E staining, oil red O staining, and immunohistochemistry assays of IL-6. Representative images were captured. Scale bar, 200 μm. N = 5–6 mice/group. * *p* < .05, ** *p* < .01, *** p < .001, compared with HFC control mice

We then evaluated the therapeutic effect of *A. muciniphila* in treating MAFLD in an obese mouse model induced by HFC diet feeding (Figure 1a). Compared with the HFC group mice, mice treated with *A. muciniphila* efficiently increased the abundance of *A. muciniphila* in the gut by 20 folds compared with that in HFC mice by quantitative polymerase chain reaction (qPCR) (Figure 1b). This activity led to decreased mouse body weight, as indicated by an approximately 20.8% body weight reduction in *A. muciniphila*-treated HFC mice after 6 weeks of treatment (Figure 1c). Intriguingly, *A. muciniphila* treatment efficiently attenuated hepatic steatosis in HFC mice indicated by decreased hepatic TG (Figure 1d). Diacylglycerol (DAG) is a representative intermediate of lipid metabolism that induces activation of oxidative stress and displays toxicity to hepatocytes, leading to apoptosis and liver injury *in vivo*.^26^ Notably, treatment with *A. muciniphila* in obese mice markedly decreased the DAG levels in the liver (Figure 1e). Determination of plasma levels of liver-specific enzymes revealed that *A. muciniphila* significantly ameliorated liver injury in HFC mice, as indicated by decreased levels of aspartate aminotransferase (AST), alanine aminotransferase (ALT) and alkaline phosphatase (ALP) (Figure 1f). Additionally, pathologic examination further confirmed the therapeutic potency of *A. muciniphila* in ameliorating all the phenotypes of MAFLD in mice, namely hepatocyte injury and ballooning (indicated by H＆E staining), hepatic steatosis (indicated by oil red O (ORO) staining), and inflammation (indicated by IL-6 and CD68 examination) (Figure 1g). Notably, this therapeutic effect was not due to the altered feeding behavior (Figure S2A). Gene expression analysis revealed that *A. muciniphila* markedly suppressed the expression levels of genes related to steatosis and inflammation at the transcriptional level, such as tumor necrosis factor ɑ (*TNF-ɑ*), interleukin-6 (*IL-6*), monocyte chemotactic protein 1 (*MCP-1*), fatty acid synthase (*FAS*), and acetyl CoA carboxylase (*ACC*) (Figure 1h).

revised figure 1

To explore whether these anti-MAFLD effects of *A. muciniphila* were drug-treatment dependent, the HFC mice were treated with *A. muciniphila* orally for 6 weeks, followed by the withdrawal of *A. muciniphila* for another 4 weeks (Figure 1i). Interestingly, *A. muciniphila* withdrawal caused efficient persistence of its anti-MAFLD activities, as indicated by significant reductions in the mouse body weight (Figure 1j), hepatic TG levels (Figure 1k), and plasma levels of AST, ALT, and ALP (Figure 1l) in the post-drug withdrawal group. Pathologic examination further confirmed the conclusion that *A. muciniphila* withdrawal caused sustained anti-MAFLD activity in mice (Figure 1m). These maintained therapeutic effects in *A. muciniphila*-treated HFC mice were correlated with higher abundance of *A. muciniphila* (Figure S2B-F).

We further determined the anti-MAFLD properties of *A. muciniphila* in antibiotic (Abx)-treated HFC mice (Figure S3A). Treatment with Abx in mice efficiently decreased the abundance of *A. muciniphila* and its colonization in gut (Figure S3B). This activity led to an exacerbation of liver injury and MAFLD manifestation in mice, as indicated by increased liver weight, heavy hepatic TG accumulation, and liver injury in Abx-treated HFC mice (Figure S3C-G). Additionally, there were still significant MAFLD-attenuating effects in the Abx and *A. muciniphila* co-treatment group compared with that in the HFC mice or Abx-treated HFC mice. Gene expression analysis further confirmed that the ability of *A. muciniphila* in downregulating the gene expression levels of *FAS, ACC, TNF-ɑ, IL-6*, and *MCP1* (Figure S3H). The above data demonstrate that treatment with *A. muciniphila* efficiently alleviates MAFLD in mice.

### *A. muciniphila* activated lipid oxidation in the gut-liver axis and maintains gut barrier integrity

**Figure 2.**
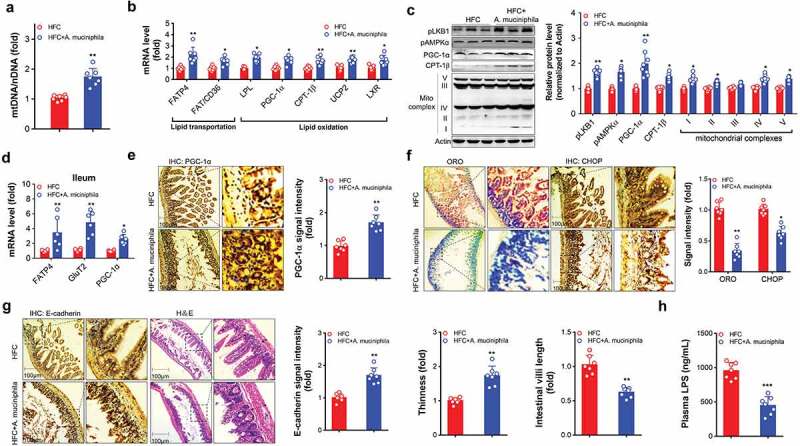
*A. muciniphila* increased markers related to lipid metabolism in liver and gut of HFC mice. HFC induced obese MAFLD (11 weeks of feeding) were administered with saline as the control or A. *muciniphila* (6 weeks of treatment) to assess liver and ileum parameters: a-c for parameters in the liver and d-f for parameters in the ileum. e-g, representative images of the ileum (Scale bar, for 100 µm). (a) Hepatic mitochondrial copy number determination. (b) Gene expression related to lipid uptake and oxidation in the liver of mice. (c) Protein levels of metabolic regulators mitochondrial complexes, and the LKB1-AMPK axis in the liver of mice. The protein levels were quantified and normalized to the loading control actin (d) Gene expression of lipid uptake and oxidation in the ileum of mice. The gene level or protein level in the HFC group was set as 1, and the relative fold increases were determined by comparison with the HFC control group. (e) Immunohistochemistry analysis of PGC-1α in the ileum of mice. The brown dot indicates the examined protein. Representative images were captured. Scale bar, 100 µm. (f) TG level quantification (indicated by oil red O staining) and oxidative stress-induced cell apoptosis determination (indicated by CHOP examination) in the ileum. Representative images were captured. Scale bar, 100 µm. (g) Immunohistochemistry analysis of E-cadherin and H＆E examination in ileum of HFC mice. The brown dot indicates the target protein. Representative images were captured. Scale bar, 100 µm. N = 5–8 mice/group. * *p* < .05, ** *p* < .01, *** p < .001, compared with HFC control mice

Studies have reported that treatment with *A. muciniphila* increases lipid oxidation and activates energy expenditure, which are the foundations underlying its therapeutic properties in obesity.^16,19^ Consistently, mice treated with *A. muciniphila* showed increased mitochondrial copy numbers (Figure 2a) and activated a network of genes involved in lipid transportation [fatty acid transport protein 4 (*FATP4*), fatty acid translocase/cluster of differentiation 36 (*FAT/CD36*)] and oxidation [lipoprotein lipase (*LPL*), peroxisome proliferator-activated receptor γ coactiva-tor-1ɑ (*PGC-1ɑ*), uncoupling protein 2 (*UCP2*), carnitine palmitoyltransterase-1β (*CPT-1β*), and liver X receptor (*LXR*)] in the liver (Figure 2b). Immunoblotting assays revealed that treatment with *A. muciniphila* in mice activated the liver kinase B1 (LKB1)-AMPK axis, and increased the expression of energy metabolic regulators (PGC-1ɑ and CPT-1β) and mitochondrial complexes (I, II, IV, and V) in liver (Figure 2c).

revised figure 2

Consistent with the beneficial metabolic profile in the liver, mice treated with *A. muciniphila* also activated a network of genes involved in glucose and lipid transportation (indicated by *FATP4* and *GluT2*) and oxidation (indicated by *PGC-1α*) in the ileum and colon, respectively (Figure 2d, Figure S4A). Immunohistochemistry analysis of PGC-1α in ileum and colon further confirmed the metabolic stimulation effects of *A. muciniphila* (Figure 2e, Figure S4B). These improved metabolic effects led to the reduction of the lipid levels (indicated by ORO staining) and attenuated oxidation stress-induced cell apoptosis in ileum and colon [indicated by C/EBP-homologous protein (CHOP) protein examination] (Figure 2f, Figure S4C), thus improving the integrity of the gut barrier, as indicated by examination of the marker for cell tight connection (E-cadherin) and the thickness of intestinal mucosal by H＆E staining (Figure 2g, Figure S4D). Shorten villi length in ileum was observed in the HFC mice treated with *A. muciniphila* (Figure 2g). Furthermore, determination of plasma lipopolysaccharide (LPS) levels in HFC mice further supports the result that treatment with *A. muciniphila* can improve the gut integrity of gut barrier since remarkable decreased plasma LPS levels in *A. muciniphila*-treated HFC mice was observed (Figure 2h).

### *A. muciniphila* attenuated bile acid metabolism dysfunction in the gut-liver axis in obese mice

*In vivo*, bile acid is mainly synthesized in the liver, transported to the tissues of gut and gallbladder, and then refluxed to the liver.^27^ Its transportation is tightly regulated by a network of genes, including bile acid synthesis-related genes [e.g., cholesterol 7α-hydroxylase (*CYP7A1*), 7-alpha-hydroxycholest-4-en-3-one 12-alpha-hydroxylase (*CYP8B1*), and sterol 27α-hydroxylase (*CYP27A1*)], bile acid transportation-related genes [e.g., G-protein coupled bile acid receptor 1 (*TGR5*), bile salt export pump (*BSEP*), multidrug resistance associated protein 2/3 (*MRP2/3*)], bile acid salt reflux regulatory genes [apical sodium-dependent bile salt transporter (*ASBT*), ileal bile acid-binding protein (*IBABP*), organic solute transporter β (*OSTβ*), Na^+^/taurocholate co-transporting polypeptide (*NTCP*), and organic anion co-transporting polypeptides (*OATP*)].^28^ Studies have shown that clinical obese and MAFLD populations often demonstrate abnormal levels of bile acid metabolism, such as high levels of bile acid metabolites in the gut and plasma.^29,30^ Moreover, high levels of bile acids in the gut are harmful to the survival of gut microbiota, leading to the development of chronic metabolic disorders.^31,32^

**Figure 3.**
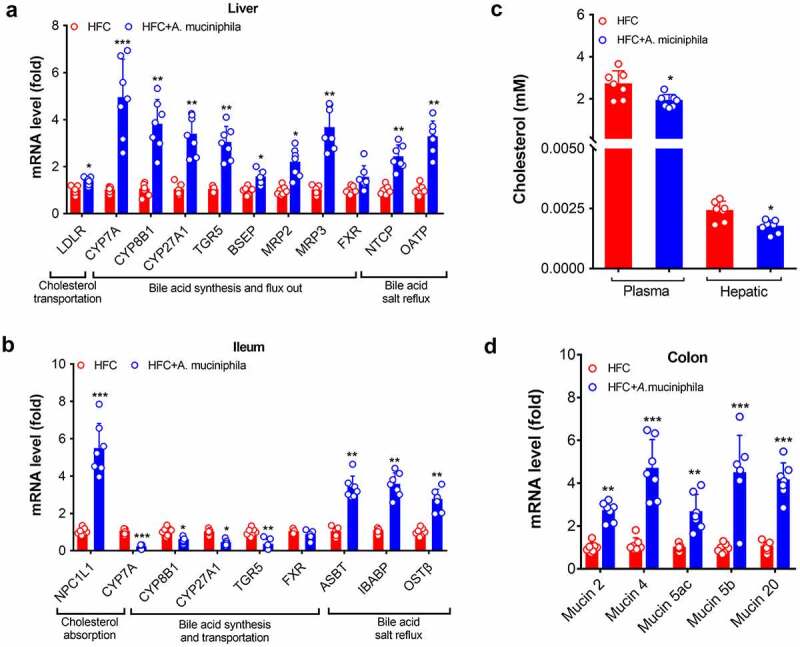
*A. muciniphila* promoted the metabolism of bile acid in the liver and gut of HFC mice. HFC-induced obese MAFLD (11 weeks of feeding) were administered saline as the control or A. *muciniphila* (6 weeks of treatment) to assess the liver and ileum parameters. (a) Schematic diagram of bile acid shuttling between the liver and gut. (b) Expression of mRNAs involved in the regulation of the synthesis and transport of bile acids in the liver. (c) Expression of mRNAs involved in the regulation of the synthesis and transport of bile acids in the ileum (d) Plasma level of cholesterol in plasma or the liver. (e) mRNA levels of mucoprotein-related genes in the colon of HFC mice. The level of genes in the HFC control group were set as 1, and the relative fold increases were determined by comparison with the HFC control group. N = 5–8 mice/group. * *p* < .05, ** *p* < .01, *** p < .001, compared with the HFC control group mice

We then explored the modulating effect of *A. muciniphila* on bile acid metabolism in the gut-liver axis. As shown in Figure 3a, mice treated with *A. muciniphila* facilitated cholesterol transport in the liver indicated by an increase in the expression levels of genes involved in cholesterol transportation-related genes in the liver, such as low-density lipoprotein receptor (*LDLR*), and activated a network of genes involved in bile acid synthesis and transportation. Additionally, gavage of *A. muciniphila* in HFC mice increased the expression levels of bile acid reflux regulatory genes in gut and liver, such as *ASBT, IBABP, OSTβ, NTCP* and *OATP* (Figure 3a-b). Furthermore, *A. muciniphila* treatment enhanced cholesterol transportation [indicated by the increased level of the cholesterol transporter Niemann-Pick type C1-like intracellular cholesterol transporter 1 (*NPC1L1*)] and decreased expression levels of bile acid synthesis-related genes in ileum (Figure 3b). Consistent with the altered expression levels of cholesterol transportation regulator (*LDLR*), HFC mice treated with *A. muciniphila* decreased the plasma and hepatic cholesterol levels (Figure 3c), suggesting that *A. muciniphila* is beneficial for bile acid metabolism in gut-liver axis.

revised figure 3

Additionally, mice treated with *A. muciniphila* showed increased levels of *mucin 2, mucin 4, mucin 5ac, mucin 5b*, and *mucin 20* in colon (Figure 3d), genes that encode mucoproteins that are key substrates for *A. muciniphila* growth and colonization in gut. This result is consistent with previous reports.^19,33^

### *A. muciniphila* increased the hepatic L-aspartate level by facilitating L-aspartate transportation from gut

**Figure 4.**
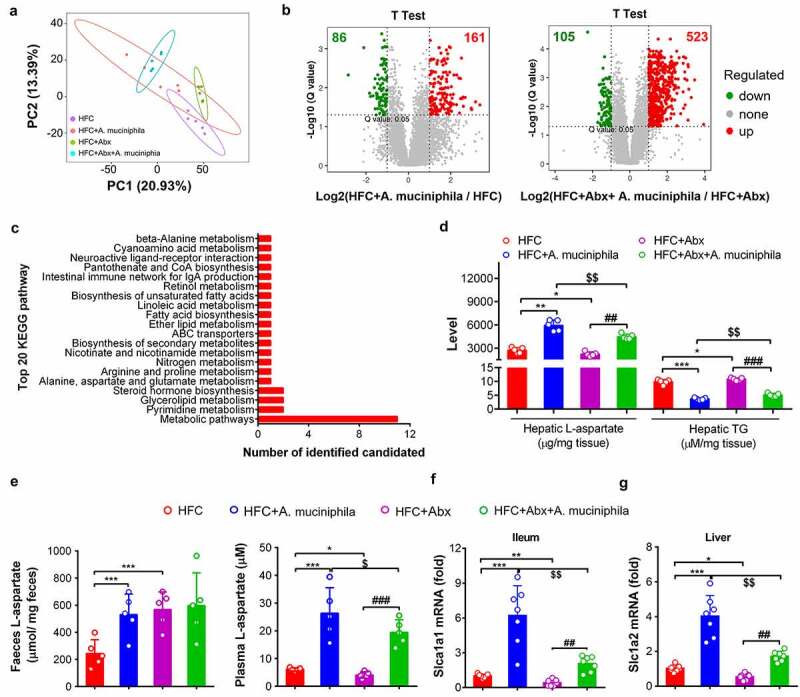
*A. muciniphila* increased L-aspartate level in liver of HFC mice. HFC-induced obese MAFLD (11 weeks of feeding) were administered saline (HFC), *A. muciniphila*, antibiotics (Abx) or a combination of Abx plus *A. muciniphila* (Abx+*A. muciniphila*) for (6 weeks of treatment) to assess liver metabolomics and the indicated assays. (a) PCA analysis. (b) Identification of significant differentially metabolites in *A. muciniphila-* or Abx+*A. muciniphila-*treated HFC mice compared with HFC or Abx-treated HFC mice by LC-MS analysis. Significant differentially metabolites were identified based on the criteria of fold ≥ 2 and a Q value equal to or higher than 0.05. The dots represent differentially metabolites. N = 5 mice/group. (c) Annotation of the identified significant metabolites in the HFC, HFC+*A. muciniphila*, HFC+Abx, and HFC+Abx+*A. muciniphila* groups by metabolomics trend analysis. (d) Comparative analysis of the hepatic L-aspartate and TG levels. (e) L-aspartate level in the feces and plasma of mice. N = 5 mice/group. (f) Gene expression of *Slc1a1* in the ileum of mice. (g) Gene expression of *Slc1a2* in the liver of mice. The level of mRNA in the HFC control group was set as 1, and relative fold increases were determined by comparison with the HFC control group. N = 5–8 mice/group. * *p* < .05, ** *p* < .01, *** p < .001, compared with HFC control mice. # *p* < .05, ## *p* < .05, ### *p* < .001, compared with antibiotics (Abx)-treated HFC mice; ^$^ *p* < .05, ^$$^ *p* < .01, ^$$$^ *p* < .001, compared with *A. muciniphila*-treated HFC mice

To reveal the possible mode of action underlying the anti-MAFLD activities of *A. muciniphila*, we determined the metabolic profile in liver by performing metabolomics analysis. Principal coordinates analysis (PCA) revealed significant differences in the chemical component in HFC+*A. muciniphila* (compared with HFC group), HFC+Abx (compared with HFC group), and HFC+Abx+*A. muciniphila* (compared with HFC+Abx group) (Figure 4a). Compared with HFC mice, mice treated with *A. muciniphila* displayed a different metabolism profile, as indicated by the detection of 247 metabolites with significant differences (86 downregulated and 161 upregulated) (Figure 4b**, left panel**). The identified metabolites were assigned to categories according to the Kyoto Encyclopedia of Genes and Genomes (KEGG) database. In total, 98 metabolites were classified into the top 20 KEGG second-level pathways (Figure S5A). Additionally, more significantly different metabolites were detected in the livers of the combination group of *A. muciniphila* and Abx compared with those in HFC mice treated with Abx alone. In total, 628 significantly different metabolites were detected (105 downregulated and 523 upregulated) (Figure 4b**, right panel**). These altered metabolites were annotated to be correlated with hepatic metabolism, such as lipolysis, fat digestion and absorption, insulin resistance, and bile acid biosynthesis (Figure S5B). Next, we performed metabolic trend analysis among these groups. Intriguingly, several interesting metabolites were identified, such as aspartic acid, corticosterone, acylcarnitine, and trans-retinoic acid, *etc*, and were assigned to categories according to the KEGG database (Figure 4c). Among them, L-aspartate was identified by occupying a higher significance level (~2.18-fold) and a lower *p* value (*p* = .0000114) than that in HFC mice (Table S2), correlated with most of the annotated top 20 KEGG pathways, such as metabolic pathway, pyrimidine metabolism, glycerolipid metabolism, alanine, aspartate, and glutamate metabolism, arginine and proline metabolism, and nitrogen metabolism. Quantification of hepatic L-aspartate level revealed that *A. muciniphila* treatment significantly increased the hepatic L-aspartate levels while reduced hepatic L-aspartate levels were detected in Abx-treated HFC mice (Figure 4d). Additionally, Abx treatment partly abrogated the stimulation effect of *A. muciniphila* in increasing the hepatic L-aspartate levels compared with *A. muciniphila* treatment alone. Furthermore, the hepatic L-aspartate levels are negatively correlated with the hepatic TG content (Figure 4d), indicating that *A. muciniphila* ameliorating MAFLD could be related with L-aspartate, which is a potential agent for MAFLD treatment.^34^

revised Figure 4

We further determined the levels of L-aspartate in plasma and feces. *A. muciniphila* treatment efficiently increased the L-aspartate levels in plasma and feces while Abx treatment also increased the L-aspartate level in feces but decreased the level of L-aspartate in the plasma and liver (Figure 4d-e). Consistently, gene expression of the L-aspartate transporters (*Slc1a1 or Slc1a2*)^35,36^ in ileum or liver revealed that mice treated with *A. muciniphila* increased the expression levels of *Slc1a1* or *Slc1a2* while Abx treatment decreased the expression levels of L-aspartate transporters and partially abolished the stimulation effect of *A. muciniphila* in increasing the gene expression of the L-aspartate transporter (Figure 4f-g).

### L-aspartate directly activated lipid oxidation and the LKB1-AMPK axis

**Figure 5.**
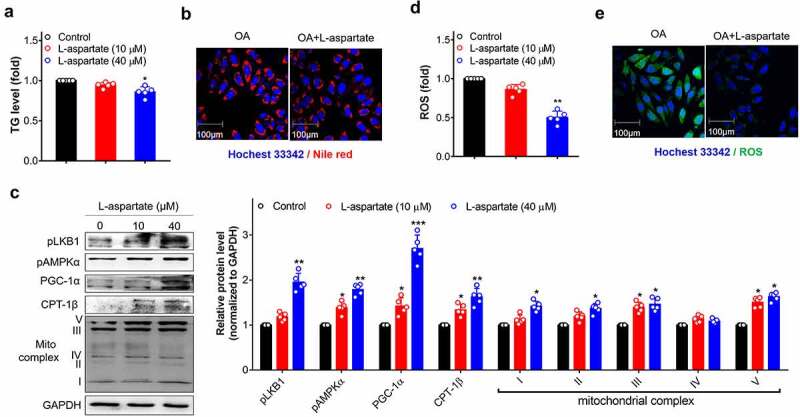
L-aspartate reduced lipid and ROS in hepatocytes along with increases in LKB1-AMPK activity and mitochondrial oxidative capacity. L-02 cells induced with oleatic acid (0.75 mM) were treated with L-aspartate (10, 40 µM) for 24 h, and then cells were harvested for the indicated analysis. (a) TG level in cell lysates. (b) Nile red staining using a Nile red probe at a final concentration of 5 μM. (c) Key proteins in the LKB1-AMPK axis and mitochondrial oxidation. The protein levels were quantified against the loading control GAPDH. The protein levels in control cells of each independent experiment were set as 1, and the relative fold increases were determined by comparison with the control group. (d-e) Quantification of the ROS levels by flowcytometry or confocal microscopy using the DCFH-DA probe at a final concentration of 10 μM. Representative images were captured. Scale bar, 100 µm. The data shown are individual values with means ± SEM. N = 5 independent experiments. * *p* < .05, ** *p* < .01, *** p < .001, compared with control group cells

We exposed L-aspartate to hepatocytes (L-02 cell) in the presence of oleatic acid (OA) induction, a well-known reported cell model that mimics hepatic steatosis *in vitro*^6^ and found that the addition of L-aspartate efficiently decreased the cellular lipid levels, as indicated by the TG assay and Nile red staining (Figure 5a-b). This reduction occurred with increased expression levels of metabolic energy regulators (PGC-1α) and mitochondrial complexes and activation of the LKB1-AMPK axis (Figure 5c), leading to inhibition of oxidative stress, as indicated by a decrease in the levels of reactive oxygen species (ROS) (Figure 5d-e). This beneficial metabolic effect was also replicated in OA-inducted intestinal cells (Figure S6). These novel findings agreed well with the latest report revealing the metabolic role of L-aspartate in activating the LKB1-AMPK axis in HCT-116 cells.^37^

To further unravel the direct metabolic role of L-aspartate *in vivo*, mice were orally treated with L-aspartate for 24 h, and the result showed that the expression of the gene for the L-aspartate transporter was efficiently activated at the transcriptional level, leading to the increase of L-aspartate levels in liver (Figure S7A-B). Additionally, L-aspartate treatment activated a network of genes correlated with hepatic energy oxidation, such as *PGC-1α, UCP2*, and *CPT-1β* (Figure S7C). Immunohistochemistry analysis of PGC-1α and UCP2 further confirmed the direct metabolic beneficial effect of L-aspartate in liver (Figure S7D). These metabolic improvements resulted in a slight reduction in the endogenous hepatic TG level without liver injury (Figure S7E).

We also explored the modulating effect of L-aspartate on bile acid metabolism in the gut-liver axis. Interestingly, L-aspartate treatment markedly activated a network of genes involved in bile acid synthesis and transportation in the gut-liver axis (Figure S5F-G). These data further confirmed the direct metabolic effects of L-aspartate in regulating energy metabolism, such as lipid oxidation and bile acid metabolism.

We then explored the therapeutic effect of L-aspartate in MAFLD. As expected, gavage with L-aspartate (200 mgkg^-1^) in HFC mice efficiently prevented liver weight increase and ameliorated liver injury and hepatic steatosis (Figure S8A-B). Quantification of hepatic TG levels and determination of plasma liver-specific enzyme levels (AST, ALT, ALP) in HFC mice further confirmed the anti-MAFLD activity of L-aspartate *in vivo* (Figure S8C-D), suggesting that L-aspartate could be an efficient agent for MAFLD treatment.

## Discussion

The present study investigated the therapeutic effects of *A. muciniphila* in MAFLD and its underlying molecular mode in a HFC diet-induced obese mouse model by different interventions. Our study showed that obese mice treated with *A. muciniphila* eliminated hepatic steatosis, inflammation, and liver injury. These therapeutic effects largely fulfilled the recommended criteria essential for the treatment of MAFLD.^9^ Interestingly, these anti-MAFLD actions of *A. muciniphila* in mice also persisted after the withdrawal of *A. muciniphila* treatment or the administration of *A. muciniphila* after antibiotics treatment. Notably, *A. muciniphila* treatment facilitated L-aspartate transportation to liver from the gut, which further activated LKB1-AMPK axis and stimulated lipid oxidation, thus ameliorating MAFLD in mice. Furthermore, treated with *A. muciniphila* in mice enhanced cholesterol transportation and ameliorated abnormal bile acid metabolism in gut-liver axis. These metabolic effects could be beneficial to reshape gut microbiota composition. Our study provided a new clue in explaining the metabolic effect of *A. muciniphila*. To our knowledge, this is the first study to verify the beneficial role of L-aspartate in combating MAFL and the novel role of *A. muciniphila* in regulating L-aspartate metabolism.

*A. muciniphila*, the next-generation beneficial microbes, is severely reduced in several pathological conditions (such as obesity, type 2 diabetes, inflammatory bowel diseases, and appendicitis)^38,^^39,40^ Either direct oral administration of *A. muciniphila* or enrichment of the abundance of *A. muciniphila in vivo* through dietary or pharmacologic interventions has been proved to be a practicable therapeutic strategy for metabolic disorders including amelioration in body weight and fat mass, improvement in insulin-resistance and liver fat accumulation.^16,17,19^ A latest study revealed that *A. muciniphila* was decreased in patients with alcoholic liver disease (ALD) and recovery of *A. muciniphila* in ALD mice protects against hepatic steatosis and injury.^21^ Enriched abundance of *A. muciniphila* in MAFLD phenotype obese mice protected liver from all manifestations of MAFLD, further implying that *A. muciniphila* would be an efficacy agent for liver disease therapy. Consisted with latest findings, our study further confirmed the beneficial therapeutic effect of *A. muciniphila* in MAFLD. Interestingly, the withdrawal of *A. muciniphila* treatment *in vivo* for 4 weeks still maintained its therapeutic effect due to the reshaping gut microbiota. This long-lasting amelioration effect may greatly benefit the clinical application of *A. muciniphila* in the future. Moreover, we found that administration of *A. muciniphila* in antibiotics-treated HFC mice marginally weakened the anti-MAFLD effect of *A. muciniphila* with less *A. muciniphila* colonization in gut. Although this novel finding still needs to be further proved, it still led us to wonder the underlying mechanism with a great interest.

To date, several studies have been performed to try to reveal the mechanism of how *A. muciniphila* regulates energy expenditure and signal transduction for its metabolic effects. Among which, a study done by Zhao *et al* revealed that *A. muciniphila* supplementation reduced fatty acid synthesis and transport in liver and muscle with reduced chronic low-grade inflammation through inactivating LPS/LBP downstream signaling.^17^ Also, administration of pasteurized *A. muciniphila* in mice alleviated diet-induced obesity and decreased food energy efficiency through a reduction of carbohydrates absorption and enhanced intestinal epithelial turnover.^41^ Notably, an outer-membrane protein of *A. muciniphila*, termed ‘Amuc_1100ʹ, was identified, which interacted with toll-like receptor 2 to improve the gut barrier and partly recapitulate the beneficial effects of the bacterium.^19^ However, the detailed mechanisms correlating *A. muciniphila* (in gut) with its therapeutic effect in target tissues are still unknown. Our study demonstrated that mice treated with *A. muciniphila* increased levels of several metabolites in liver including SCFAs and L-aspartate. Quantification of L-aspartate levels revealed that *A. muciniphila* treatment increased the levels of L-aspartate in the feces, plasma and liver of mice. This stimulating effect of *A. muciniphila* in promoting L-aspartate transportation was further confirmed by detecting L-aspartate transporters. It is worth noting that administration of antibiotics in mice also increased L-aspartate levels in feces while reduced levels of L-aspartate in plasma and liver. Additionally, administrated antibiotics together with *A. muciniphila* efficiently abrogated the stimulating effect but a higher level of plasma L-aspartate was still detect compared with that in antibiotics-treated mice alone, indicating that gut microbiota (not just *A. muciniphila*) may play important role in regulating the production and transportation of L-aspartate. This novel finding was quite inconsistent with the traditional theory that L-aspartate being mainly produced in the liver.^42,43^ In addition to L-aspartate, we also observed that *A. muciniphila* treatment elevated the levels of SCFAs in liver including propionate and acetate, which have beneficial metabolic effects in curbing obesity and MAFLD.^44,45^ The elevated metabolites may separately or together underlie the therapeutic effect of *A. muciniphila* in MAFLD. At least, the increased L-aspartate in liver mediated by *A. muciniphila* would provide a reasonable explanation for its anti-MAFLD as demonstrated in our current study. This interesting data provided novel clues for explaining the therapeutic effect of *A. muciniphila in vivo*.

L-aspartate is a non-essential amino acid and involved in treatment for acute and chronic liver hepatitis and cirrhosis treatment in the clinic by regulating nitrogen metabolism, nucleic acid synthesis and the Kreb′s cycle.^43,46^ A recent study speculated that L-aspartate can be an efficacious therapeutic agent in treating MAFLD/NASH.^34^ However, this hypothesis has not been verified *in vivo* and *in vitro*. Recently, a study demonstrated that L-aspartate supplementation could ameliorate diabetic kidney disease in mice.^47^ In mechanism, a latest study done by Jiang *et al* revealed that L-aspartate can directly interact with phosphorylated LKB1 leading to an activation of LKB1-AMPK axis in cells.^37^ Consistently, our study also found L-aspartate treatment activated LKB1-AMPK axis in hepatocyte and intestinal cells, enhanced lipid oxidation and inhibited oxidative stress and its induced cell injury, thus ameliorating MAFLD. Moreover, a negative correlation was observed between the L-aspartate level and the hepatic TG level, suggesting that increased L-aspartate level in liver may present a therapeutic strategy for MAFLD. This finding further underlies the conclusion of our study that *A. muciniphila* ameliorated MAFLD in mice by regulating L-aspartate metabolism. In the future, we will verify the effectiveness of L-aspartate in the treatment of MAFLD in human subjects and further explore the novel practicable interventions that can elevate the L-aspartate level in the liver for MAFLD treatment.

On the other hand, although our study also verified the therapeutic effect of *A. muciniphila* through activating lipid oxidation, we observed some inconsistent results with others, including 1) *A. muciniphila* treatment increased lipid and cholesterol transportation in the ileum and liver, 2) a shorten villi in ileum of *A. muciniphila*-treated HFC mice was observed, and shorten villi often indicates decreased ability to absorb nutrient, which is inconsistent with our gene results. To our knowledge, the difference of these results might be partly due to a combination of reasons. First, our current study of administrated mice with a HFC diet, cholesterol is critical to induce liver injury and metabolic syndrome with robust nonalcoholic steatohepatitis. High daily cholesterol intake induced oxidative stress in the ileum and dysfunction bile acid metabolism between liver-gut axis, leading to thinning of the intestinal wall (as indicated by H＆E examination). Treatment with *A. muciniphila* in mice facilitated gut–liver interaction and cholesterol absorption and metabolism, thus maintained the integrity of gut barrier. Second, L-aspartate is an important oxidative fuel that can enter mitochondria for ATP synthesis to absorb nutrients.^48^ High levels of L-aspartate in the ileum and liver of obese mice can accelerate lipids and cholesterol absorption and oxidation. Indeed, either restriction of energy absorption or promotion of energy oxidation has been proved to be an efficacy strategy for metabolic disorders treatment.^6,49^ The shorten villi may be the result of a complicated consequence of high abundance of *A. muciniphila* and the reshaped gut microbiota composition in the gut, and high intake of cholesterol.

Dysfunction of the gut-liver axis is closely correlated with the development of MAFLD.^50^ Among activities in the gut-liver axis, bile acid metabolism dysfunction plays pivotal roles in reshaping the gut microbiota composition and regulating the homeostasis of glucose and lipids in liver.^51^ Studies have revealed that MAFLD or NASH populations is often accompanied by abnormal bile acid level in plasma, which are positively related to the degree of MAFLD.^29,30^ Moreover, high-fat and cholesterol diet feeding alters the composition of bile acids in the gut, causing imbalances in the intestinal flora and aggravating bile acid metabolism disorders.^52^ In contrast, enhanced bile acid metabolism is beneficial to ameliorate MAFLD.^53,54^ Our study revealed that *A. muciniphila* treatment increased cholesterol absorption and bile acid metabolism in the liver, and improved the recycling of the bile acid metabolism in the gut-liver axis. The enhanced bile acid metabolism might be beneficial to remodel gut microbiota composition and the colonization of *A. muciniphila*.^55^ Additionally, recent studies have demonstrated that the activation of oxidative stress and induction of cell apoptosis in the gut are harmful to the gut barrier, leading to the development of certain disease, such as inflammatory bowel disease (IBD), obesity and MAFLD.^56–58^ Consistently, *A. muciniphila* treatment efficiently increased lipid oxidation, ameliorated accumulated lipids induced oxidative stress and cell apoptosis in gut and maintained the integrity of the gut barrier. These data further confirmed the therapeutic effects of *A. muciniphila* in treating metabolic disorders.

The development of MAFLD involves multiple hits.^59^ Among them, hepatic steatosis is recognized as the initial factor, characterized by heavy lipid accumulation originated from increased *de novo* lipogenesis and/or excessive flux of exogenous FFAs.^60^ Hepatic lipogenesis is regulated by a series of lipogenic enzymes, including ACC and FAS, while FFA absorption is regulated by its transporters, including FAT/CD36 and FATP4.^61^ Our results showed that *A. muciniphila* treatment not only significantly downregulated the expression of lipogenic markers but also increased the levels of FAT/CD36 and FATP4 in the liver, leading to an oxidation of accumulated lipids. These data suggest that *A. muciniphila* eliminates hepatic steatosis in the liver of HFC mice through a mechanism by increasing lipid oxidation. This mechanism greatly accords with the proved therapeutic strategy in MAFLD by enhancing hepatic lipid oxidation.

The present study has a few limitations. (i) We did not reveal the mechanism of L-aspartate in treating MAFLD in the HFC diet-induced obese mouse model. Based on its beneficial metabolic role in cells and in regular diet mice, we reasonably believe that L-aspartate ameliorates MAFLD in mice closely related to its metabolic regulation effect. (ii) We did not provide clear clues to prove where the increased L-aspartate originated from. (iii) Lastly but not important, our study administrated the phosphate buffered saline (PBS) as a vehicle has a certain limitation, ideally another bacterium or a mixture of other bacteria should be selected. This needs further study to explore its anti-MAFLD actions and origin using relevant animal model.

**Figure 6.**
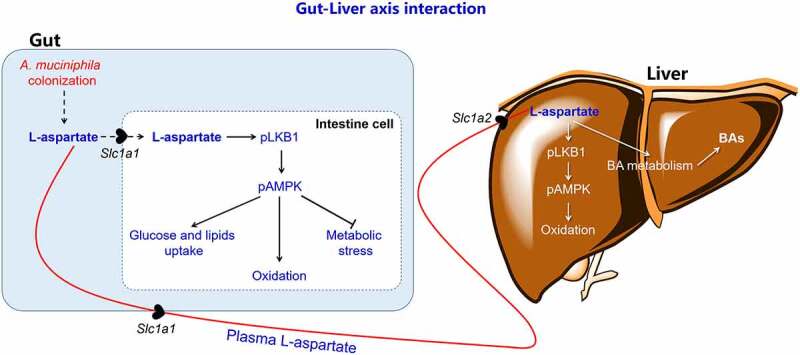
Proposed mechanism by which *A. muciniphila* ameliorates MAFLD in HFC diet-induced obese mice. *A. muciniphila* treatment increases L-aspartate levels in the gut-liver axis, which activates the LKB1-AMPK axis and increases lipid oxidation and bile acid metabolism in the gut-liver axis, leading to decreased lipid accumulation and oxidation stress-induced cell apoptosis. These metabolic improvements result in maintaining the integrity of the gut barrier and reshaping the gut microbiota composition for MAFLD treatment in mice

In summary, our present study showed that *A. muciniphila* is a potential therapeutic agent for MAFLD treatment through a novel mechanism by increasing the L-aspartate levels in liver transported from gut. Treatment with L-aspartate in cells and in mice largely replicated the beneficial metabolic effects of *A. muciniphila* in mice, including increased lipid oxidation, activation of the LKB1-AMPK axis and improved bile acid metabolism in the gut-liver axis, leading to the amelioration of lipid accumulation-induced metabolic disorders in the tissues of gut and liver (Figure 6**/Graphic abstract**). Our findings suggest that *A. muciniphila* attenuates MAFLD in mice by improving the gut–liver interactions *via* regulating L-aspartate metabolism.

revised 6

## Materials and methods

### Materials

L-aspartate (Sigma, O7125), Hochest 33342 probe (Thermo, Cat# 62249), Nile red probe (Sigma, N3013), Mito-tracker (Thermo, M7512), TG assay kit (Jiancheng Bio Cat# A110‐2) and DAG assay kit (Uscn Life Science Inc Cat# CEC038Ge), L-aspartate ELISA kit (SinoBestBio, YX-12010 M), FAS antibody (Cell Signaling Technology Cat# 3180, RRID:AB_2100796), ACC antibody (Cell Signaling Technology Cat# 3662, RRID:AB_2219400), IL-6 antibody (Cell Signaling Technology Cat# 12912, RRID:AB_2798059), TNF-α antibody (Affinity Biosciences Cat# AF7014, RRID:AB_2835319), pLKB1 antibody (Santa Cruz Biotechnology Cat# sc-271924, RRID:AB_10610759), PGC-1α antibody (Abcam Cat# ab191838, RRID:AB_2721267), CPT-1β antibody (Affinity Biosciences Cat# DF3904, RRID:AB_2836257), mitochondrial complex antibody, CD68 antibody (Abcam# ab955; RRID: AB_307338). CHOP antibody (Affinity Biosciences Cat# DF6025, RRID:AB_2838000), and E-cadherin antibody (Affinity Biosciences Cat# AF0131, RRID:AB_2833315), TRIzol reagent (Invitrogen, Cat# 15596018), cDNA synthesis kit (Takara, Cat# 6210B). Details of the primer sequences used in the study are shown in Supporting Information.

### *Akkermansia muciniphila* culture

*Akkermansia muciniphila* strain (ATCC BAA-835) was cultured in modified brain-heart infusion (BHI) liquid medium at 37 °C under anaerobic conditions. The following were added to the standard BHI (37 g/L, BD, LOT: 8114937) base ingredients: mucin (2 g/L, Sigma, CAS# 84082–64-4) and L-cysteine (5 mg/L, Sigma, CAS# 7048–04-6) (10% H_2_, 10% CO_2_, 80% N_2_).^62^

### Animal experiments

All animal care and experimental procedures were approved by the Sun Yat‐sen University Committee on Animal Ethics for the Use of Laboratory Animals in accordance with the Animal Welfare Legislation of China. Every effort was made to minimize the use of the animals and their discomfort. Animal studies are reported in compliance with the ARRIVE guidelines.^63,64^ Male C57BL/6 mice (IMSR Cat# JAX:000664, RRID:IMSR_JAX:000664) aged 7–8 weeks (18–20 g) bred at the Laboratory Animal Center of Sun Yat‐sen University (Guangzhou, China) were used for the study. The mice were housed under specific pathogen free and reared in line with standardized methods at 22 ± 1°C on a 12‐hr light/dark cycle with free access to food and water.

After 1 week of acclimatization to the environment of this study, the mice were fed with a 60% high fat and 1.2% cholesterol (HFC) diet (ResearchDiet, D12492) ad libitum for up to 17 weeks, and HFC fed mice were randomly divided into two subgroups at the beginning of week 11 to receive phosphate buffered solution (to exclude the impact of it solvent, PBS, control group) or *A. muciniphila* treatment (treatment group) for 6 weeks. The *A. muciniphila* was dissolved in oxygen-free PBS and orally each other day with a final concentration of 1 × 10^8^ CFU per mL. Suspension solution (200 μL) was given every other day. The control subgroup mice were administered the same volume of vehicle.

For the germ-free mice model, HFC mice were orally fed with antibiotics mixture [Abx containing ampicillin (0.2 g/L, Sigma, CAS# 7177–48-2), vancomycin (0.2 g/L, Sigma, CAS# 1404–90-6), neomycin sulfate (0.2 g/L, Sigma, CAS# 1405–10-3) and metronidazole (0.2 g/L, Sigma, CAS# 443–48-1)] for 1 week prior of week 11 and then received the treatment of Abx or *A. muciniphila* alone for another 6 weeks. The Abx was dissolved in drink water. Mouse body weight were monitored daily.

### Plasma biochemistry analysis

At the end of the study, mice were fasted for 8 h and anaesthetized by an *i.p*. injection of 80 mg·kg^−1^ ketamine and 10 mg·kg^−1^ xylazine. When the mice were fully anaesthetized, the eyeball was removed to collect blood samples in a tube containing 1 mM EDTA for the measurement of hepatic relevant enzymes, such as alkaine phosphatase (ALP), aspartate aminotransferase (AST) and alanine aminotransferase (ALT).

### Determination of hepatic triglyceride (TG) and diacylglycerol (DAG) levels

Liver TG and DAG were extracted and determined as described previously.^6^ Briefly, the liver tissue was weighted, homogenized and lysis. The supernatant was collected and extracted with equal volumes of chloroform/methanol. The chloroform phase was removed to a new tube and dried and was then re‐suspended in isopropyl alcohol as a total lipid extract sample. The quantities of total TG and DAG in the livers were then assayed according to the manufacturers’ protocols.

### Histological examination

The interested tissues were weighed and subjected to oil-red O staining or fixed in 4% formaldehyde solution, then embedded in paraffin after dehydration in a graded ethanol series (70–100%). Embedded samples were sectioned (4 μm thick) with a rotary microtome (Leica, Germany) and subject to hematoxylin and eosin (H & E). The liver macrophages were assessed by immunohistochemistry labeled with CD68 and IL-6. To determine the metabolism, apoptosis and the integrity of gut barrier in gut, the level of protein peroxisome proliferator-activated receptor γ coactivator-1α (PGC-1α), C/EBP-homologous protein (CHOP), and E-cadherin were examined. Representative images were captured and quantification analysis was performed in 10 randomly selected fields per sample in a blinded manner as our previously reported.^6^

### 16S rRNA sequencing amplicon

The bacterial DNA was extracted from fecal samples with a QIAamp Fast DNA stool Mini Kit (Qiagen, Cat# 51604) and PCR amplification was conducted with barcoded specific bacterial primers targeting the variable region 3–4 (V3–V4) of the 16S rRNA gene: forward primer 5′-ACTCCTACGGGAGGCAGCA-3′ and reverse primer 5′-GGACTACHVGGGTWTCTAAT-3′.^65^ Construction of sequencing libraries and paired-end sequencing was performed on an Illumina MiSeq platform at Biomarker Technologies Co, Ltd. (Beijing, China) according to standard protocols. Raw sequences were merged and quality filtered, and duplicates were removed by FLASH, Trimmomatic, and UCHIME, respectively.^66^ The resulting sequences were then aligned against the Greengenes database of 16S rRNA gene sequences. Reads were then performed using quantitative insights into microbial ecology (QIIME) analysis. Raw sequences were deposited in the Sequence Read Archive database (http://www.ncbinlm.nih.gov/sra), with the accession numbers ranging from SAMN16252123 to SAMN16252158.

### Total RNA extraction and real time quantitative-PCR

Total RNA from cells or tissues were isolated using the TRIzol method. The first‐ strand cDNA was synthesized with a cDNA synthesis kit. Quantitative real‐time PCR was carried out using 2 × RealStar Green Fast Mixture with ROX (GenStar, Cat# A301). The results were analyzed on an ABI StepOnePlus real‐time PCR system (Applied Biosystems, USA, RRID: SCR_015805) using the 2^−ΔΔCt^ method as described previously.^67^ Primers were designed by Primer Premier 5, synthesized by Generay Biotech (Guangzhou, China), and listed in (Table S1). Actin and 16S rRNA were used as a loading control, and normalized the relative mRNA levels.

### Immunoblotting and immunofluorescence

Immunoblotting and immunostaining were performed as previously described.^6^ Briefly, cells or tissues were lysed and total protein were extracted and quantified. Proteins were subjected to SDS-PAGE and transferred to PVDF membrane, and blocked with 5% bovine serum albumin (BSA) – Tris-buffered saline Tween for 30 min at room temperature, then membrane were incubated with primary antibodies overnight and followed secondary antibody incubation, protein bands were visualized with an ECL kit (Millipore). The uncropped data of immunoblotting were uploaded as a supplemented file. For Immunostaining, cells were fixed and blocked, then incubated with primary antibodies at 4°C overnight. After washing, cells were co-stained with fluorescent labeled secondary antibody and 2 μg/mL DAPI (for nucleus) at 37°C for 1 h. Fluorescence images were acquired using confocal microscope (Zeiss, Germany) with a 60× UPlanApoN oil immersion lens (NA 1.40).

### Hepatic metabolomics analysis

The metabolites in the livers were extracted with 50% methanol Buffer. 20 μL of sample was extracted with 120 μL of precooled 50% methanol, vortexed for 1 min, and incubated at room temperature for 10 min; the extraction mixture was then stored overnight at −20°C. After centrifugation at 4,000 g for 20 min, the supernatants were transferred into new 96-well plates for LC-MS analysis. In addition, pooled QC samples were also prepared by combining 10 μL of each extraction mixture. These samples were then subjected to LC-MS system followed machine orders. A high-resolution tandem mass spectrometer TripleTOF5600 plus (SCIEX, UK) was used to detect metabolites eluted from the ACQUITY UPLC BEH Amide column (100 mm × 2.1 mm, 1.7 µm, Waters, UK). The Q-TOF was operated in both positive and negative ion modes. The curtain gas was set 30 PSI, Ion source gas1 was set 60 PSI, Ion source gas2 was set 60 PSI, and an interface heater temperature was 650°C. For positive ion mode, the ionspray voltage floating were set at −4500 V, respectively. The mass spectrometry data were acquired in IDA mode. The TOF mass range was from 60 to 1200 Da. The survey scans were acquired in 150 millisecond and as many as 12 product ion scans were collected if exceeding a threshold of 100 counts per second (counts/s) and with a 1+ charge-state. Total cycle time was fixed to 0.56 s. Four time bins were summed for each scan at a pulser frequency value of 11 kHz through monitoring of the 40 GHz multichannel TDC detector with four-anode/channel detection. Dynamic exclusion was set for 4 s. During the acquisition, the mass accuracy was calibrated every 20 samples. Furthermore, in order to evaluate the stability of the LC-MS during the whole acquisition, a quality control sample (Pool of all samples) was acquired after every 10 samples.

### Mitochondrial copy number quantification

Quantification of mitochondrial DNA (mtDNA) copy numbers was achieved by PCR. Briefly, DNA was extracted from cells or liver tissue using a DNeasy Blood and Tissue kit (Tiagen Biotech, China). The copy numbers of nuclear DNA (nDNA) and mtDNA were assessed by PCR targeted toward the cytochrome C gene (for mtDNA) and 18S rRNA (for nDNA).

### L-aspartate level determination

The levels of L-aspartate in liver, feces and plasma were measured by an L-aspartate ELISA kits according to the manufacturer’s instructions. The feces and liver samples were dissolved and sonicated in saline buffer and centrifuged at 1,000 g for 10 min at 4°C to remove debris. Briefly, 20 μL samples, saline, and standard was added to the ELISA plate and incubated at 37°C for 1 h. Wells were washed four times with elution buffer and then incubated with biotin‐labeled antibodies at 37°C in the dark for another 30 min followed by reaction with the corresponding substrate. The color produced was proportional to the concentration of L-aspartate was measured at 450 nm and quantified against the standard curve from the known amount of the cytokine.

### Metabolic flux assay

Cellular metabolic rates were measured using a XF96 Analyzer (Seahorse Bioscience, USA). L-02 cells were treated with vehicle or L-aspartate for 24 h, the ECAR and OCR were determined by a Seahorse Bioscience XF96 Extracellular Flux Analyzer (Seahorse Bioscience, USA), with 2 μM oligomycin, 1.5 μM FCCP and 1 μM antimycin A/rotenone injected during fixed time intervals. The OCR were normalized by protein level in each well. Mitochondrial respiration of BAT isolated from mice was determined using an XF24 Extracellular Flux Analyzer (Seahorse Bioscience, USA) using 5 μg mitochondrial protein in a buffer containing 50 mM KCl, 4 mM KH_2_PO_4_, 5 mM HEPES, and 1 mM EGTA, 4% BSA, 10 mM Pyruvate, 5 mM Malate, 1 mM GDP. Mitochondria were isolated using a mitochondria isolation kit (Thermo, China), plated and centrifuged at 2,000 g for 20 min to promote adherence to the cell culture microplate. One millimole of ADP, 4 mM Oligomycin, 6 mM FCCP, and 2 mM each of Antimycin A/Rotenone were added during fixed time intervals.

### Statistical analysis

The data and statistical analysis comply with the recommendations of the British Journal of Pharmacology on experimental design and analysis in pharmacology.^68^ Cell experiment included 5 independent experiment for each data set and 8 to 10 mice were included in each group of animal study, the results were presented as the mean ± SEM unless stated otherwise. Data are expressed as the mean ± SEM. Differences between two groups were analyzed by Student’s t-test using Graphpad Prism (Graphpad Software Inc, California, USA, RRID:SCR_002798). Statistical analysis for multiple groups was performed by one‐way ANOVA followed by Tukey’s HSD post hoc tests. A *P* value of ≤ .05 was considered statistically significant.

## Supplementary Material

Supplemental Material
