# Supplementary material for: Gut Akkermansia muciniphila ameliorates metabolic dysfunction-associated fatty liver disease by regulating the metabolism of L-aspartate via gut-liver axis
Source: Gut Microbes. 2021 May 25;13(1):1927633. doi: 10.1080/19490976.2021.1927633 (PMC8158032; doi:10.1080/19490976.2021.1927633)
Supplement: Supplemental Material [file KGMI_A_1927633_SM6815.pdf]

Supporting Information

For

Gut *Akkermansia muciniphila* ameliorates metabolic dysfunction-associated fatty liver disease by regulating the metabolism of L-aspartate *via* gut-liver axis

Yong Rao[a,†,*], Zhiqi Kuang[b,c,†], Chan Li[a], Shiyao Guo[a], Yaohao Xu[a], Dandan Zhao[a], Yutao Hu[a], Bingbing Song[a], Zhi Jiang[a], Zhenhuang Ge[b,c], Xiyuan Liu[b,c], Chengdao Li[b,c], Shuobin Chen[a], Jiming Ye[d], Zhishu Huang[a,*] Yongjun Lu[b,c,*],

[a]School of Pharmaceutical Sciences, Guangdong Provincial Key Laboratory of New Drug Design and Evaluation, Sun Yat-sen University, Guangzhou, China; [b]Run Ze Laboratory for Gastrointestinal Microbiome study, School of Life Sciences, Sun Yat-sen University, Guangzhou China; [c]Biomedical Center of Sun Yat-sen University, Guangzhou, China; [d] Lipid Biology and Metabolic Disease Research Group, School of Health and Biomedical Sciences, RMIT University, Melbourne, VIC, 3083, Australia

[†] These authors contributed equally to this work.

* CONTACT

Yongjun Lu, **Email:** luyj@mail.sysu.edu.cn, Run Ze Laboratory for Gastrointestinal Microbiome study, School of Life Sciences, Sun Yat-sen University, Guangzhou, China.

Zhishu Huang, **Email:** ceshzs@mail.sysu.edu.cn, School of Pharmaceutical Sciences, Sun Yat-sen University, Guangzhou, China.

Yong Rao, **Email:** raoyong0805@126.com, School of Pharmaceutical Sciences, Sun Yat-sen University, Guangzhou, China.

Contents

**Table S1**	Primers sequence information	S3-S5
**Table S2**	Representative significant metabolites were identified in the liver of *A. muciniphila*-treated HFC mice	S6
**Figure S1**	*A. muciniphila* reshaped the gut microbiota composition in HFC mice	S7
**Figure S2**	Withdrawal of *A. muciniphila* treatment maintained the reshaped gut microbiota in HFC mice	S8
**Figure S3**	*A. muciniphila* attenuated MAFLD in in antibiotics-treated HFC mice	S9
**Figure S4**	*A. muciniphila* increased lipid oxidation and maintains the integrity of the gut barrier in the colon tissue of HFC mice	S10
**Figure S5**	Annotation of the significant metabolites in the liver of *A. muciniphila*-treated HFC mice by assessing to KEGG database	S11
**Figure S6**	L-aspartate activated energy metabolic regulators and LKB1-AMPK axis in intestinal cells	S12
**Figure S7**	L-aspartate activated energy metabolism and bile acid metabolism in the gut-liver axis of mice	S13
**Figure S8**	Oral L-aspartate efficiently ameliorated MAFLD in HFC mice	S14

Table S1 Primer sequences for quantification of the following target genes by qPCR.

Target gene	Primer set	Sequence (5′ to 3′)
16S	AKK-F	GTCTCAAGCGTTGTTCGGAATCACT
	AKK2-R	CTACGCATTTCACTGCTACACCGAG
16S	BAC-F	ACTCCTACGGGAGGCAGCAGT
	BAC-R	GTATTACCGCGGCTGCTGGCAC
MCP1	Mcp1-F	GGTGGTTGTGGAAAAGGTAGTG
	Mcp1-R	GCTGACCCCAAGAAGGAA
FAS	Fas-F	GTCCCAGAAATCGCCTATG
	Fas-R	GGGTCATCCTGTCTCCTTTT
FATP4	Fatp4-F	CGTATGGCTTCCCTGGTGTA
	Fatp4-R	GCCAATCCCCACGATGTTT
FAT/CD36	Fat/cd36-F	CAGTGCGTCATCTACGGGTTG
	Fat/cd36-R	GCAGGTAGCGGCAGATTTCA
LPL	Lpl-F	GGACTGAGGATGGCAAGCA
	Lpl-R	TGAGCAGTTCTCCGATGTCC
PGC-1α	Pgc1α-F	AGAAGCGGGAGTCTGAAA
	Pgc1α-R	TCACAGGTGTAACGGTAGG
CPT-1β	Cpt1β-F	ATGTATCGCCGCAAACTG
	Cpt1β-R	TGCCTGGGATGCGTGTAGT
UCP2	Ucp2-F	CCTTCCTTTCTCCGCTTGG
	Ucp2-R	AAGGTGCCTCCCGAGATTG
LXR	Lxr-F	CCAAAATGCTGGGGAACG
	Lxr-R	GCGTGCTCCCTTGATGACA
GluT2	Glut2-F	ATGTCGGTGGGACTTGTGC
	Glut2-R	GGAAGCAGAGGGCGATGA
CYP7A1	Cyp7a1-F	TTGTTCAAGACCGCACATAAAGC
	Cyp7a1-R	CATCAAAGGTGGAGAGTGTATCGTT
CYP8B1	Cyp8b1-F	AAGGCTGGCTTCCTGAGCTT

	Cyp8b1-R	AACAGCTCATCGGCCTCATC
CYP27A1	Cyp27a1-F	GATGGCTGAGGAAGAAAGAGG
	Cyp27a1-R	ACAGTCTTTACTTCTCCCATCCC
TGR5	Tgr5-F	CAAGCCTCATCGTCATCGCCAA
	Tgr5-R	GCAAGCAGGGAAAGGAAACAAAAGT
Slc10a2	ASBT-F	ACCACTTGCTCCACACTGCTT
	ASBT-R	CGTTCCTGAGTCAACCCACAT
NTCP	Ntcp-F	GGAAGCCCAAAGGGGTGAT
	Ntcp-R	GCCAGGGTGAAGAGGTTAGACA
OATP	Oatp -F	ATGCTCGTTGGGTTGGTGC
	Oatp -R	CTGTCTGACCAAACTGCTGCTCTA
BSEP	Bsep P-F	TAGGGTTCTACAGGGGTTGG
	Bsep -R	CCAAAAGCAGCCACTGTTCG
MRP 2	Mrp 2-F	GCTGGGAGAAATGGAGAATG
	Mrp 2-R	CTGACCGCCACTGAGATTTA
MRP3	Mrp 3-F	GCGATAGCAGGAGTGGTTGA
	Mrp 3-R	CACCAAGTAGGCACCGAACC
NPC1L1	Npc1l1-F	CAGATGGAGCCGAGTTGC
	Npc1l1-R	CAGCCAGCACCAGGAAAG
OSTβ	Ostβ-F	CTGGCAGTCCTGGTGGTCAT
	Ostβ-R	TGGTGTTTCTTTGTCTTGTGGC
IBABP	Ibabp -F	CAGCAGGACGGACAGGACTT
	Ibabp -R	GGGAACTCTGCCACCACCTT
Mucin2	Muc2-F	GCCTGAAGACTGTCGTGCTGTTG
	Muc2-R	AATGTGGTAGGAGGAGGGTTGGA
Mucin4	Muc4-F	TGGCTACAAAGGCTACCACC
	Muc4-R	GCCCGAGGATGAGTAGATGG
Mucin5ac	Muc5ac-F	CAAACATCTGTGCGTCTGAAACC
	Muc5ac-R	GCATACTTTTCATTCTCCACGCTG

Mucin5b	Muc5b-F	GCCAAAAGTAGAGTGTAAGGAGAC
	Muc5b-R	GACTGTTCACCCAGGTTTCATT
Mucin20	Muc20-F	CCCGACGCATCCTACAACA
	Muc20-R	GAAGGACGTCCACTTCTTCCC
Actin	β-actin-F	GGCTGTATTCCCCTCCATCG
	β-actin-R	CCAGTTGGTAACAATGCCATGT
Collagen I	CollagenI-F	TCCTGGTCCTGCTGGCAAAGAA
	CollagenI-R	CACGCTGTCCAGCAATACCTTGA
TGF-β1	TGFβ-F	GAGCTGCTTATCCCAGATTCA
	TGFβ-R	GGCAGTGGAGACGTCAGATT
TNF-α	TNFα-F	ACCCTCAACCTCTTCTGGCTCAAA
	TNFα-R	AATCCCAGGTTTCGAAGTGGTGGT
IL-6	IL6-F	ATAGGACTGGAGATGTCTGAGG
	IL6-R	GCTTGTGGAGAAGGAGTTCATAG
Slc1a1	Slc1a1-F	CGCCACCATGAAAATCGTCC
	Slc1a1-R	ATACCCAAGGCAAAGCGGAA
Slc1a2	Slc1a2-F	TACTCCCCTCTGGGTATCG
	Slc1a2-R	CCCGTGAATGATGAGGC
LDLR	Ldl-F	TAGGCTATCTGCTCTTCACC
	Ldl-R	CTATTGTTGGTCACCTCCGT

Table S2 Representative significant metabolites were identified in liver of *A. muciniphila*-treated HFC mice.

**MS2 metabolite**	**Ratio** (*A. muciniphila* vs HFC control)	***p* value**
Linoleic acid methyl ester	10.4	0.00197
2-Arachidonoylglycerol	9.05	0.00138
1-Arachidonoylglycerol	6.06	0.0023
Monoelaidin	5.81	0.00095
8,11-Tridecadienoic acid	5.227	0.000098
2-Linoleoylglycerol	4.0	0.00234
Cysmethynil	3.95	0.00058
propionate	2.89	0.00111
acetate	2.22	0.00473
Thymine	2.19	0.00127
L-aspartic acid	2.18	0.0000114
Maltotetraose	0.47	0.00097
Melezitose	0.45	0.00012
N-Acetyl-L-glutamine	0.44	0.00058
Adenine	0.40	0.0029
Maltotetraose	0.39	0.00029

The significant metabolites were identified and annotated at MS2 level by LC-MS and assessing to the metabolites database. Each group concluded 5 mice, and the ratio were calculated by normalizing to HFC control group.

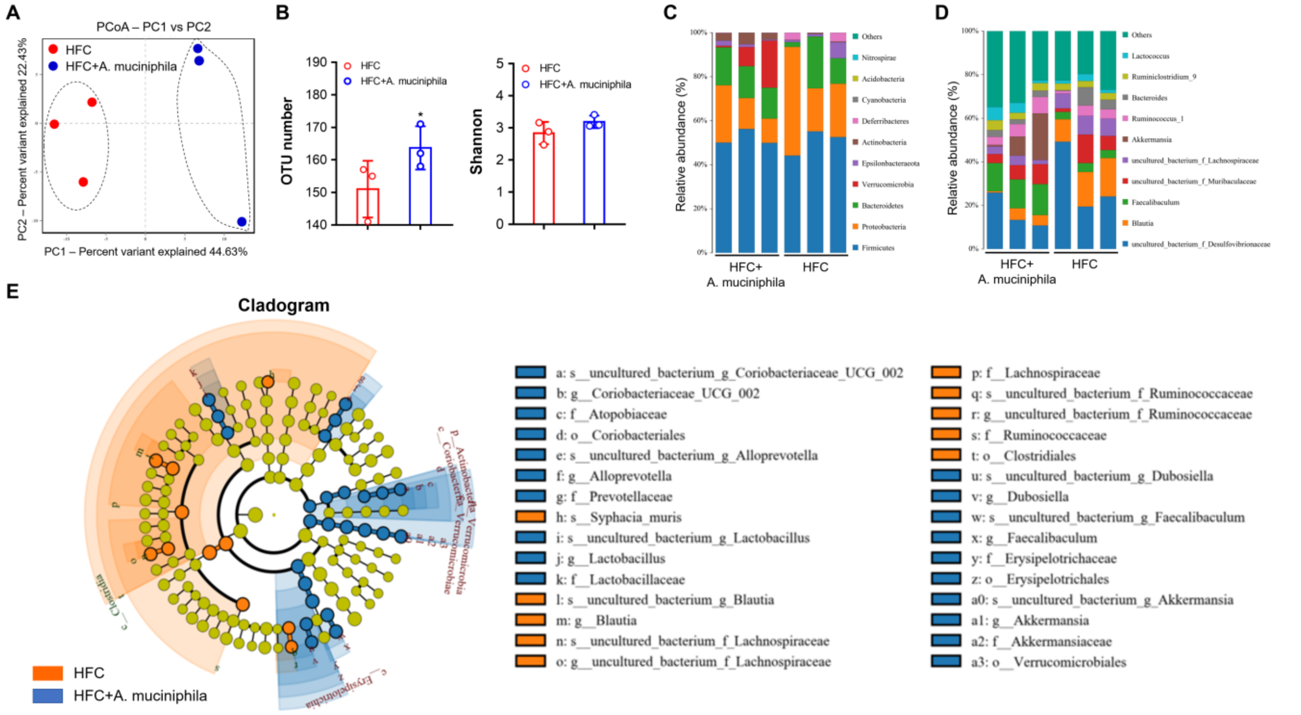

Figure S1. *A. muciniphila* reshaped the gut microbiota composition in HFC mice. HFC diet-induced obese mice (11 weeks of feeding) were administered PBS (HFC control group) or *A. muciniphila* each other day for 6 weeks. The feces were collected for the 16S rDNA amplicon sequencing assay. (A) PCoA analysis. (B) Gut microbiota species (indicated by operational taxonomic units (OTUs)) and diversity determination (indicated by the Shannon index). (C-D) Taxonomic analysis of gut microbiota at the phylum (C) and genus (D) levels. (E) The LDA score shows a significant bacterial difference between HFC and HFC+*A. muciniphila* mice. N = 5-8 mice / group, and feces samples of 3 mice were randomly selected for 16S rDNA sequencing. * p <0.05, compared with HFC control mice.

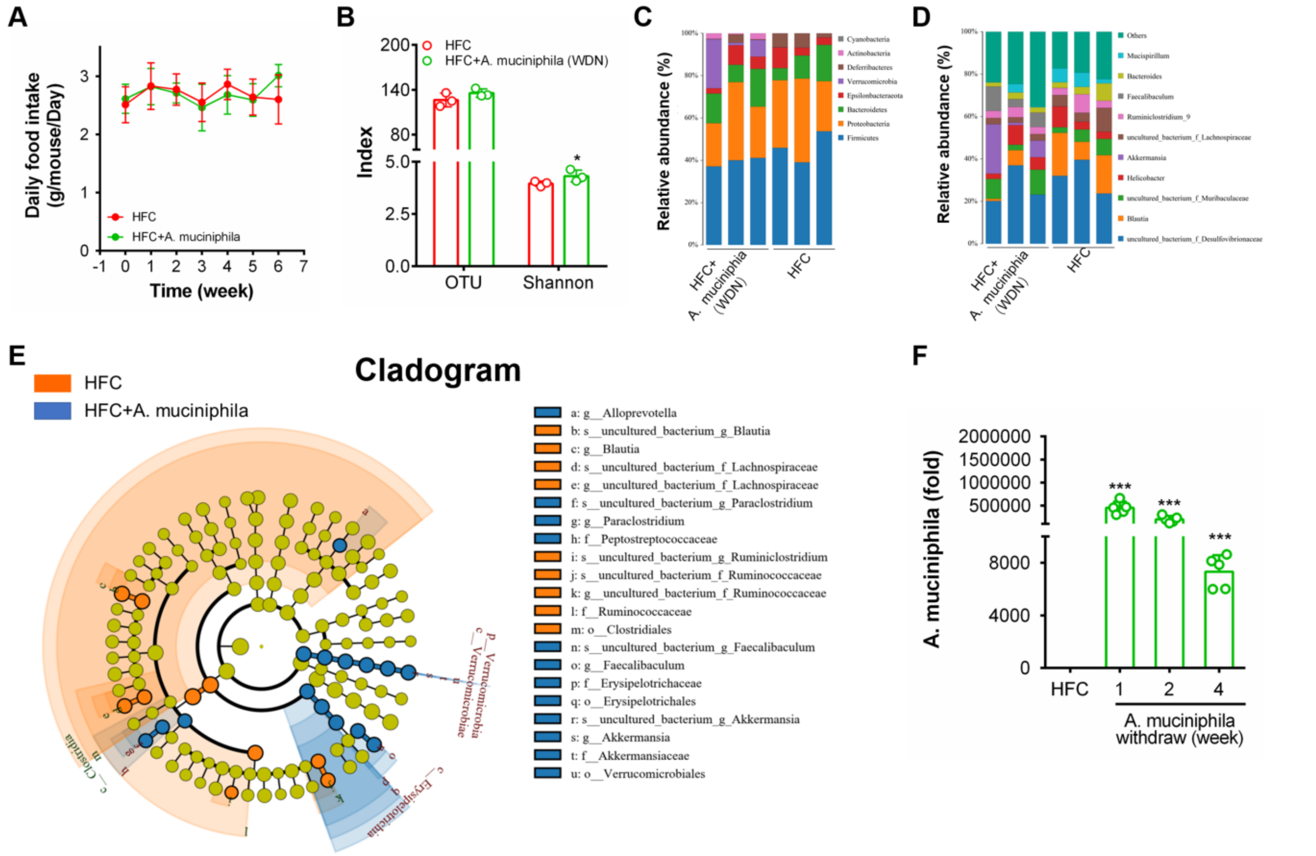

Figure S2. Withdrawal of *A. muciniphila* treatment maintained the reshaped gut microbiota in HFC mice. (A) Mouse daily food intake measurement upon *A. muciniphila* treatment for 6 weeks. (B-E) After 6 weeks treatment with *A. muciniphila*, the mice then fed with HFC diet for another 4 weeks, and feces were collected for 16S rDNA amplicon sequencing assay. (B) Gut microbiota species (indicated by operational taxonomic units (OTUs)) and diversity determination (indicated by the Shannon index). (C-D) Taxonomic analysis of gut microbiota at the phylum (C) and genus (D) levels. (E) The LDA score shows a significant bacterial difference between HFC and HFC+*A. muciniphila* (WDN) mice. N = 5 mice / group, and feces samples of 3 mice were randomly selected for 16S rDNA sequencing. (F) Determination of the abundance of *A. muciniphila* in feces of HFC mice after withdrawal *A. muciniphila* treatment. N = 5 mice / group. * p <0.05, ** p <0.01, *** p <0.001, compared with HFC control mice.

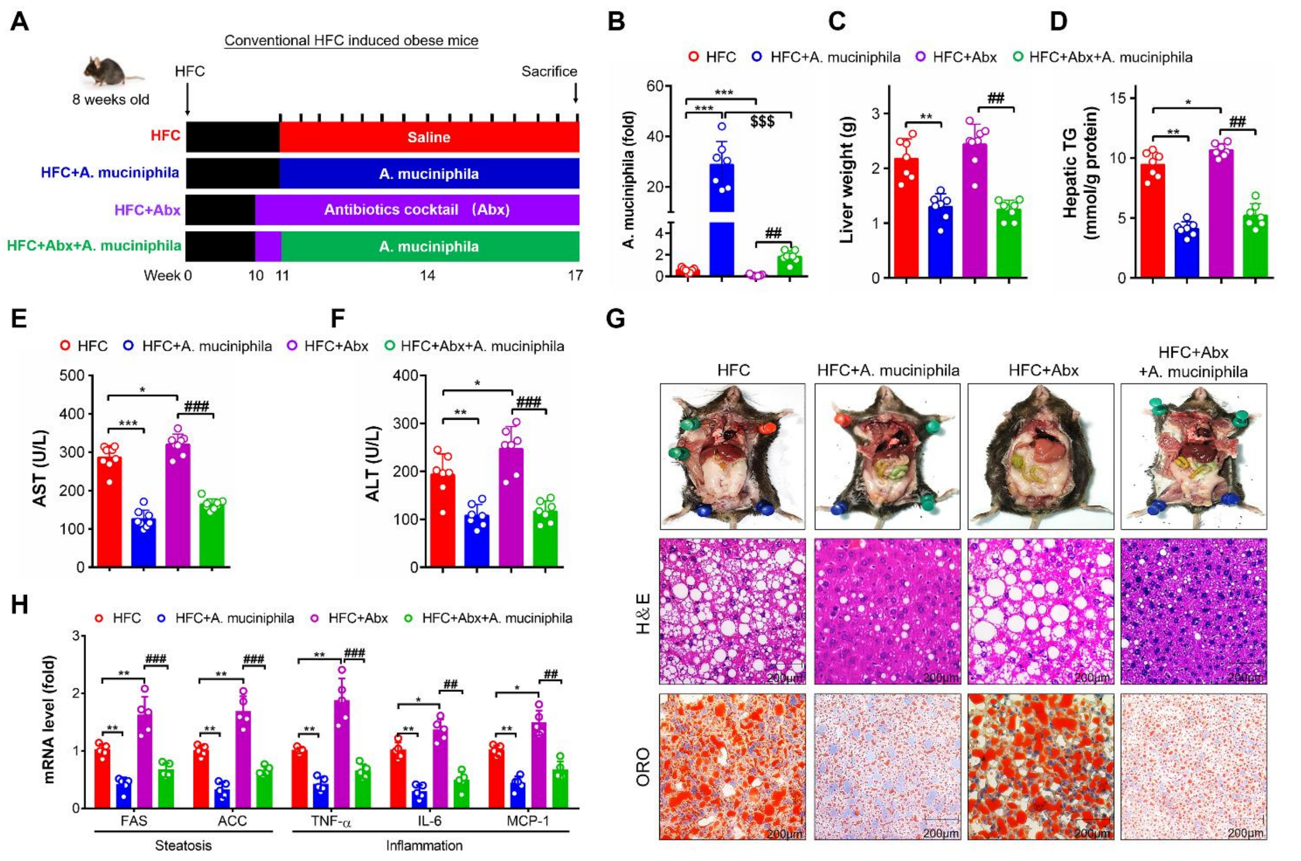

Figure S3. *A. muciniphila* attenuated MAFLD in antibiotics-treated HFC mice. HFC mice (11 weeks of HFC feeding) were treated with PBS (HFC group), *A. muciniphila* (HFC+*A. muciniphila*), and antibiotic (Abx, HFC+Abx) alone or together (HFC+Abx+*A. muciniphila*). After 6 weeks treatment, the feces, plasma and liver were collected and subjected to indicate analysis. (A) Schematic diagram of *A. muciniphila*, Abx treatment alone or together. (B) Abundance of *A. muciniphia* in feces. (C) Liver weight. (D) Hepatic TG determination. (E-F) Plasma AST and ALT levels quantification. (G) Pathologic examination of the liver by haematoxylin & eosin (H&E) staining, oil red O (ORO) staining. Scale bar, 200 µm. (H) Expression of mRNA markers for steatosis and inflammation markers. The levels of genes in the HFC control mice were set as 1, and the relative fold increases were determined by comparison with the HFC control mice. N = 5-8 mice/group. * p <0.05, ** p <0.01, *** p <0.001, compared with HFC control mice. $^{\#}$ p <0.05, $^{\#\#}$ p <0.05, compared with Antibiotics-treated HFC mice; $^{\$\$\$}$ p <0.001, compared with *A. muciniphila* treated-HFC mice.

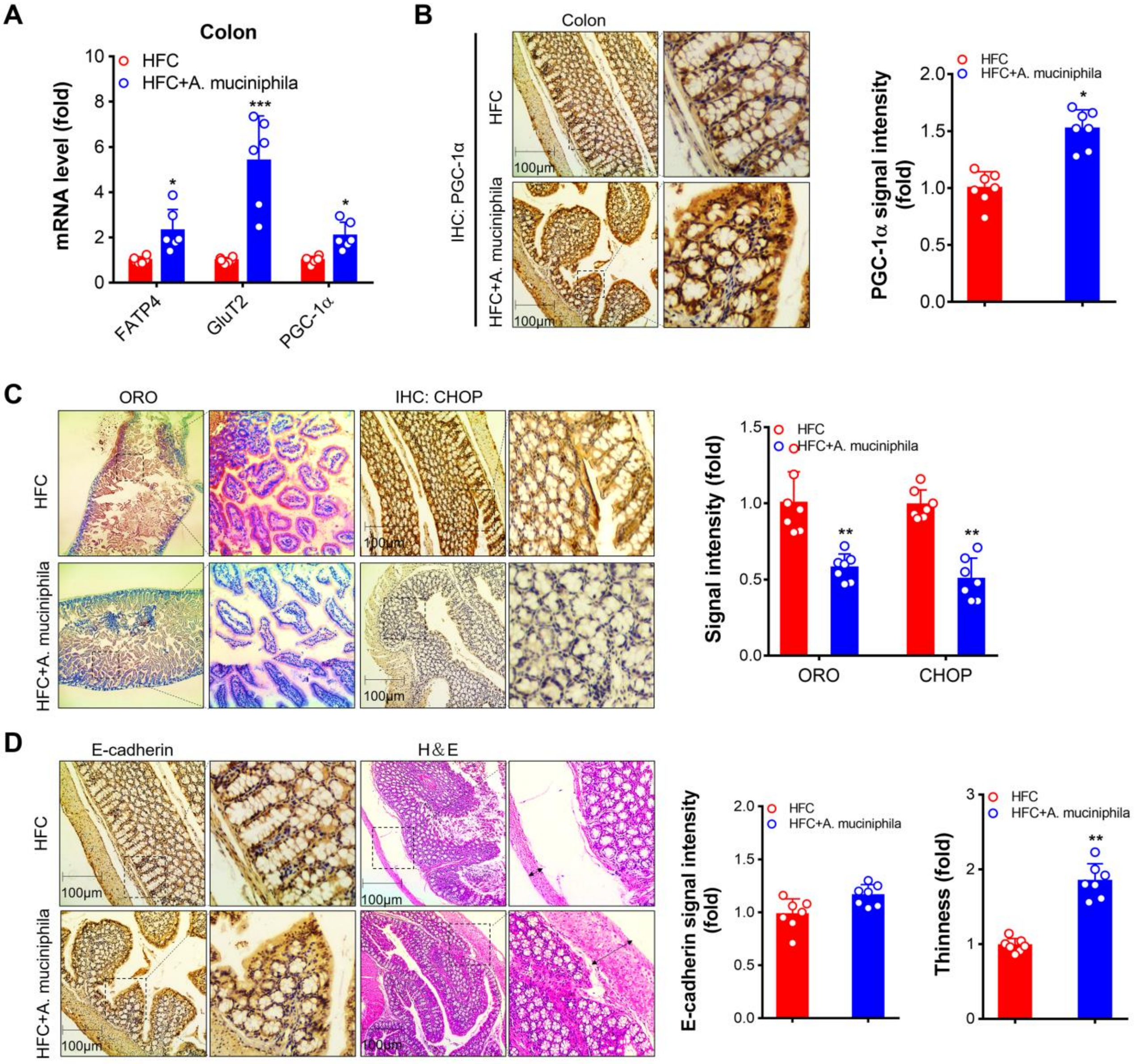

Figure S4. *A. muciniphila* increased lipid oxidation and maintains the integrity of the gut barrier in the colon tissue of HFC diet-induced obese mice. HFC diet feeding induced mice (11 weeks of feeding) were treated with PBS or *A. muciniphila* for 6 weeks, indicated metabolic parameters in the colon tissue were determined. (A) Expression of mRNA markers of lipid uptake and oxidation in the colon tissue of mice. The levels of gene in HFC control group mice were set as 1, and the relative fold increases were determined by comparison with the HFC mice. (B) Immunohistochemistry analysis of PGC-1α in the colon tissue of mice and quantification. (C) TG level quantification (indicated by ORO staining) and oxidative stress induced apoptosis (indicated by CHOP examination) determination and quantification. (D) Immunohistochemistry analysis of E-cadherin and H&E examination and quantification. The brown dot indicated the target protein. Scale bar, 100 μm. N = 5-8 mice / group. * $p < 0.05$, ** $p < 0.01$, *** $p < 0.001$, compared with HFC control mice.

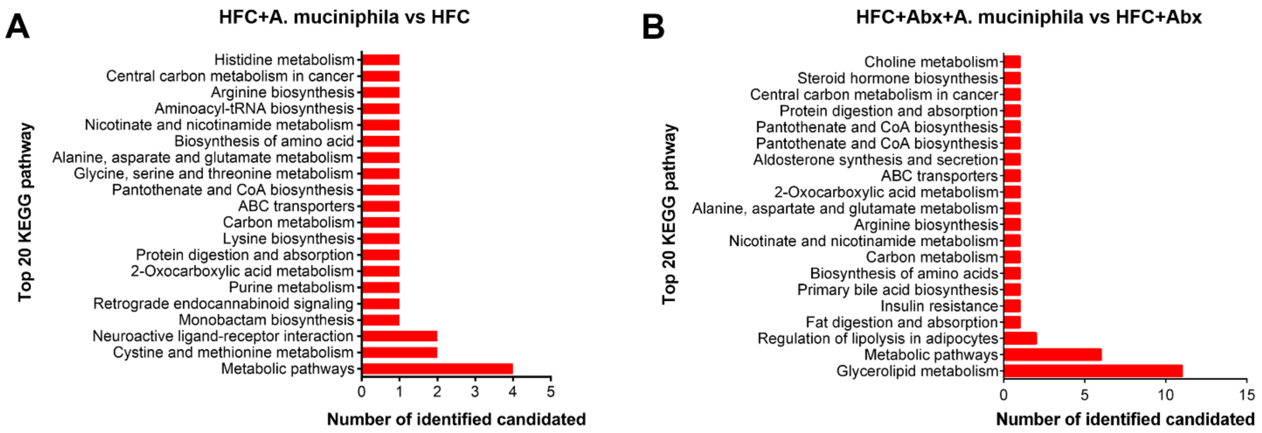

Figure S5. Annotation of the significant metabolites in the liver of *A. muciniphila*-treated HFC mice by assessing to KEGG database. The HFC diet induced obese mice (11 weeks feeding) were treated with PBS (HFC mice), Abx or *A. muciniphila* alone or together. The livers were collected at week 16 and subjected to hepatic metabolomics analysis by LC-MS analysis. (A) Biologic annotation of the significant metabolites in *A. muciniphila* treated HFC mice by assessing to the KEGG database compared with HFC mice. (B) Biologic annotation of the significant metabolites in the combination group of Abx and *A. muciniphila* treated HFC mice by assessing to the KEGG database compared with Abx-treated HFC mice. Each group included 5 mice.

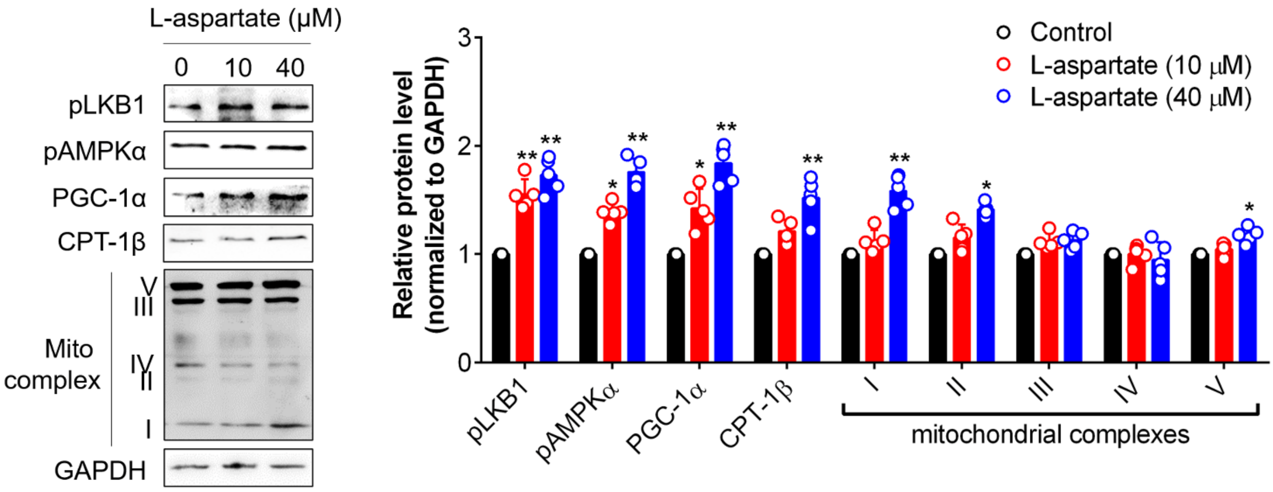

Figure S6. L-aspartate activated energy metabolic regulators and LKB1-AMPK axis in intestinal cells. NCM460 cells were treated with L-aspartate (10, 40 μM) in the presence of oleatic acid (OA, 0.75 mM) induction for 24 h. Cells were harvested and subjected to immunoblotting analysis for determining expression levels of energy metabolic regulators, mitochondrial complex and LKB1-AMPK axis. Protein levels were quantified and normalized to loading control GAPDH. The levels of protein in control cells of each independent experiment were set as 1, and the relative fold increases were determined by comparison with control cells. N = 5 independent experiments. * $p < 0.05$, ** $p < 0.01$, compared with control group cells.

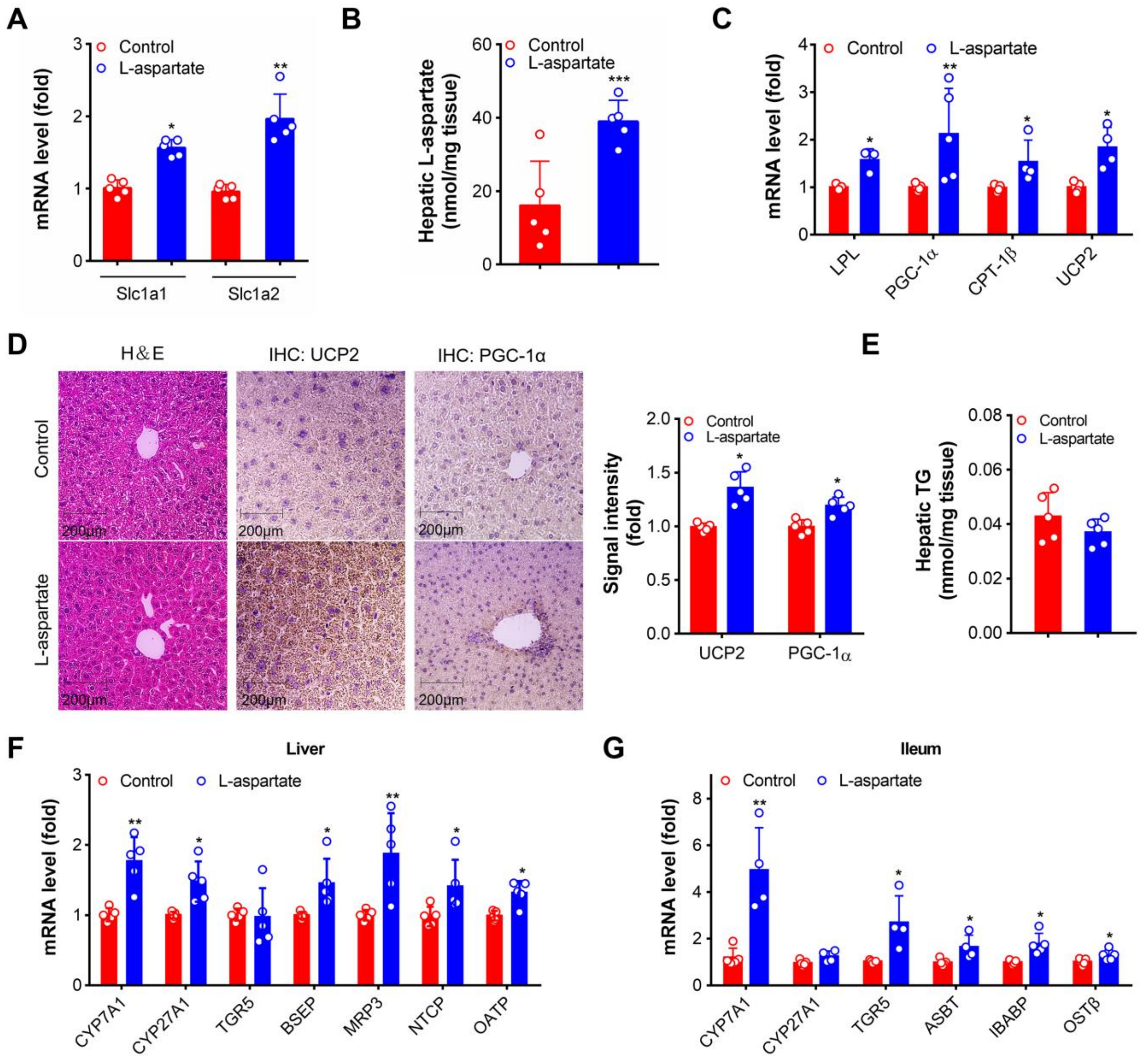

Figure S7. L-aspartate increased lipid oxidation and bile acid metabolism in the gut-liver axis of mice. Eight weeks male C57BL/6 mice were gavage with saline (control group) or L-aspartate (200 mg/kg) once for 24 h, the tissues of liver and ileum were collected and subjected to indicate analysis. (A) mRNA level of Slc1a1 and Slc1a2 in the liver of mice. (B) Determination of hepatic L-aspartate levels. (C) Expression of mRNA markers of energy metabolism regulators by qPCR assay. (D) H&E staining and immunohistochemistry analysis of PGC-1α and UCP2 in the liver and quantification. Scale bar, 200 μm. (E) Determination of hepatic TG level. (F-G) Expression of mRNA markers of bile acid synthesis and transportation in the gut-liver axis by qPCR assay. The level of genes in control mice were set as 1, and the relative fold increases were determined by comparison with the control mice. N = 5 mice /group. *$p < 0.05$, **$p < 0.01$, ***$p < 0.001$, compared with control group mice.

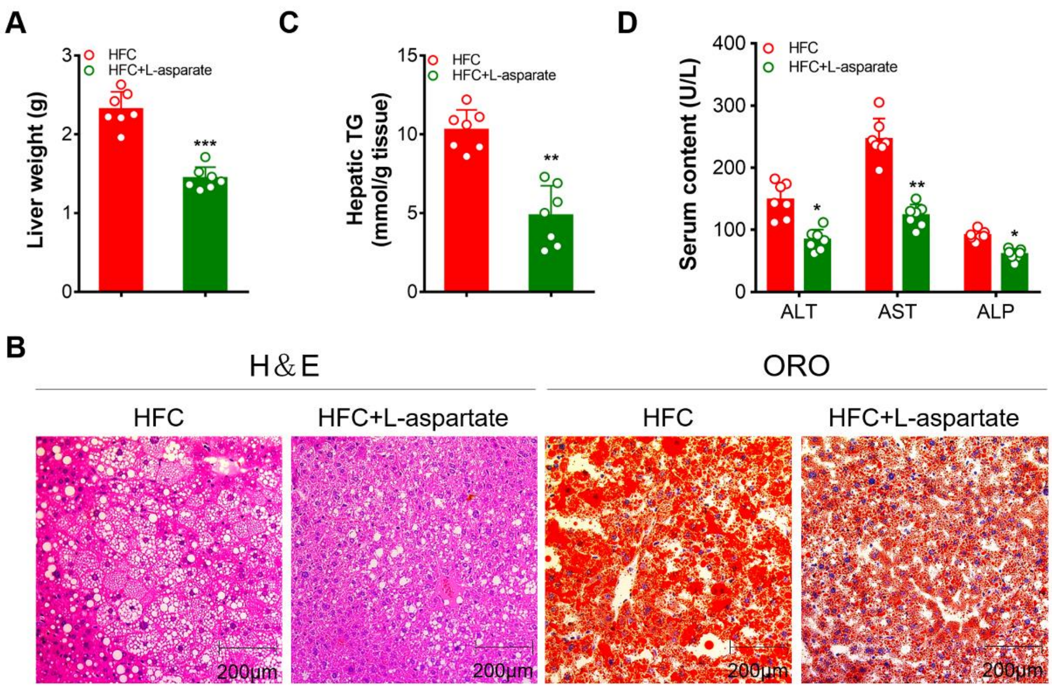

Figure S8. Oral L-aspartate efficiently ameliorated MAFLD in HFC mice. HFC diet-induced obese mice (11 weeks of feeding) were administered saline (HFC control group) or L-aspartate (200 mg/kg) orally each other day for 6 weeks. The livers were collected and subjected to indicate assays. (A) Liver weight. (B) Pathologic examination of the liver by haematoxylin & eosin (H&E) staining and ORO staining. Scale bar, 200 μm. (C) Hepatic TG level determination. (D) Determination of plasma AST, ALT, and ALP in HFC mice. N = 7 mice / group. * p <0.05, ** p <0.01, *** p <0.001, compared with HFC control mice.
